# RNA-Seq Analysis of Pubertal Mammary Epithelial Cells Reveals Novel *n*-3 Polyunsaturated Fatty Acid Transcriptomic Changes in the *fat-1* Mouse Model

**DOI:** 10.3390/nu16223925

**Published:** 2024-11-17

**Authors:** Connor D. C. Buchanan, Rahbika Ashraf, Lyn M. Hillyer, Wangshu Tu, Jing X. Kang, Sanjeena Subedi, David W. L. Ma

**Affiliations:** 1 Department of Human Health and Nutritional Sciences, University of Guelph, Guelph, ON N1G 2W1, Canada; cbucha03@uoguelph.ca (C.D.C.B.); rashraf@uoguelph.ca (R.A.); lhillyer@uoguelph.ca (L.M.H.); 2 School of Mathematics and Statistics, Carleton University, Ottawa, ON K1S 5B6, Canada; wangshutu@cunet.carleton.ca (W.T.); sanjeenadang@cunet.carleton.ca (S.S.); 3 Laboratory for Lipid Medicine and Technology, Department of Medicine, Massachusetts General Hospital and Harvard Medical School, Boston, MA 02129, USA; jxkang@oghi.org; 4 Omega-3 and Global Health Institute, Boston, MA 02129, USA

**Keywords:** *n*-3 polyunsaturated fatty acids, eicosapentaenoic acid, docosahexaenoic acid, alpha-linolenic acid, mammary epithelial cells, RNA-Seq

## Abstract

Background: The early exposure of nutrients during pubertal mammary gland development may reduce the risk of developing breast cancer later in life. Anticancer *n*-3 polyunsaturated fatty acids (*n*-3 PUFA) are shown to modulate pubertal mammary gland development; however, the mechanisms of action remain unclear. Prior work focused on effects at the whole tissue level, and little is known at the cellular level, such as at the level of mammary epithelial cells (MECs), which are implicated in cancer development. Methods: This pilot study examined the effects of lifelong *n*-3 PUFA exposure on the transcriptome by RNA-Seq in the isolated MECs of pubertal (6–8-week-old) female *fat-1* transgenic mice capable of de novo *n*-3 PUFA synthesis. *edgeR* and *DESeq2* were used separately for the differential expression analysis of RNA sequencing data followed by the Benjamani–Hochberg procedure for multiple testing correction. Results: Nine genes were found concordant and significantly different (*p* ≤ 0.05) by both the DESeq2 and edgeR methods. These genes were associated with multiple pathways, suggesting that *n*-3 PUFA stimulates estrogen-related signaling (*Mlltl0*, *Galr3*, and *Nrip1*) and a glycolytic profile (*Soga1*, *Pdpr*, and *Uso1*) while offering protective effects for immune and DNA damage responses (*Glpd1*, *Garre1*, and *Rpa1*) in MECs during puberty. Conclusions: This pilot study highlights the utility of RNA-Seq to better understanding the mechanistic effects of specific nutrients such as *n*-3 PUFA in a cell-specific manner. Thus, further studies are warranted to investigate the cell-specific mechanisms by which *n*-3 PUFA influences pubertal mammary gland development and breast cancer risk later in life.

## 1. Introduction

Breast cancer (BC) is a leading cancer diagnosed worldwide, with 2.3 million cases reported in 2022 [1] and is associated with modifiable risk factors [2]. At least 30% of cancer cases have been associated with lifestyle and dietary habits, including the intake of different types of dietary fats [2]. A number of studies have demonstrated that increasing intakes of *n*-3 polyunsaturated fatty acids (*n*-3 PUFA), which are found in fish and marine oils, may have protective effects against BC [3,4,5]. In rodent models of BC, lifelong intake of *n*-3 PUFA has been shown to reduce tumor numbers, which may be attributed to its effects on mammary gland development [4,5]. During puberty, female rodents experience extensive growth of the mammary gland driven by rapidly proliferating epithelial cells which form club-like structures known as terminal end buds (TEBs) that contain tumor initiation sites [6,7]. These highly proliferating cells are also responsible for the maturation of the ductal network and are susceptible to cancer initiation [7,8]. The intake of *n*-3 PUFA has been reported to affect pubertal mammary gland development by delaying puberty onset and reducing the number of TEBs, as well as by affecting mammary epithelial cell fate [9,10]. These findings highlight a number of effects of *n*-3 PUFA, but the precise mechanisms of action remain elusive.

To date, most studies have taken a whole-mammary-gland approach. Thus, there remains much to learn at the cellular level concerning specific cell types within the mammary gland. Recent work using cutting-edge bulk RNA sequencing (RNA-Seq) in the analysis of thousands of transcripts in tandem on isolated mammary epithelial cells has revealed cell-specific changes during the course of mammary gland development in goats [11] and in experimental mouse studies [12]. The effects of diet and *n*-3 PUFA at the cellular level have provided powerful insights into isolated mouse colonocytes [13]. However, potential novel insights into the effects of *n*-3 PUFA at the cellular level by RNA-Seq have yet to be investigated.

The *fat-1* mouse model has previously been used to study the effects of lifelong *n*-3 PUFA exposure and its mechanisms of action in many diseases, including BC [5,6], and in mammary gland development [9,10]. The *fat-1* transgene from the roundworm *Caenorhabditis elegans* encodes for *n*-3 desaturase, enabling endogenous whole-body production of *n*-3 PUFA from *n*-6 PUFA, including in the mammary gland [9,10]. This genetic approach makes the study of *n*-3 PUFA and its causal effects possible using the transgenic *fat-1* mouse model while reducing confounding effects from dietary feeding [14], thereby allowing for a precise understanding of the mechanisms of action of lifelong *n*-3 PUFA intake. Thus, this pilot study investigates the transcriptomic effects of lifelong *n*-3 PUFA exposure in mammary epithelial cells isolated from female transgenic *fat-1* mice during puberty using bulk RNA-Seq.

## 2. Materials and Methods

### 2.1. Animals, Diets, and Phenotyping

Transgenic *fat-1* mice acquired from Dr. Kang (Harvard Medical School) were used to develop an in-house breeding colony on an FVB background at the University of Guelph, as previously described [5,9,10]. The mice were fed a modified AIN93G diet (Research Diets Inc., New Brunswick, NJ, USA) containing 10% fat (*w*/*w*) from safflower oil, rich in *n*-6 PUFA, providing 22% of the mouse’s total daily energy intake requirements, as previously described [10]. In brief, safflower oil contains 70% of the essential *n*-6 PUFA linoleic acid, which transgenic *fat-1* mice will endogenously utilize to synthesize individual *n*-3 PUFA in tissues, including in the mammary gland [9,10]. Female offspring were weaned and phenotyped at three weeks of age and maintained on their parental diets until termination at 6 to 8 weeks. In rodents, puberty mammary gland development has typically been described as occurring from the age of 4 weeks up to 10 weeks [6]. To minimize the number of animals, mice mammary glands were pooled to obtain a sufficient quantity of RNA for bulk RNA Seq from epithelial cells (*n* = 3). Each sample (*n* = 1) represents the pooling of inguinal mammary glands from 2 to 3 mice. Overall, there were *n* = 3 pooled samples each for wild-type (WT) and *fat-1* mice.

### 2.2. Euthanization, Tissue Collection, Epithelial Cell Isolation, and RNA Extraction

The mice were terminated and the right and left 4th and 5th mammary glands (MGs) were excised for epithelial cell isolation, as previously described [10]. A total of 6 to 8 mice were pooled for each analysis of epithelial cells (*n* = 3); lymph nodes were removed, and mammary epithelial cells were isolated using the Prater method, as previously described [10,15]. In brief, finely minced MGs were digested in collagenase/hyaluronidase (StemCell, cat # 07912) for 18 h at 37 °C to allow for tissue dissociation. The cells were washed in Hank’s balanced salt solution (Sigma, cat # H6648) and treated with ammonium chloride (Sigma, cat # A9434), Trypsin/EDTA (Sigma, cat # T4049), dispase (StemCell, cat # 07913), and DNAase 1 (Sigma, cat # D5025) to release the epithelial cells. Five million cells were used for total RNA extraction. RNA was extracted using the Purelink RNA Mini Kit (Thermo Fisher Scientific, cat # 12183018A) following the kit instructions. The purity of the RNA was assessed using the Agilent bioanalyzer 2100 where all samples had an RNA integrity number (RIN) greater than 9 (out of a 10-point scale). RNA samples were stored at −80 °C for later analysis.

### 2.3. RNA Sequencing and RNA Sequencing Analysis

The samples were sent to the Centre for Applied Genomics in the Hospital for Sick Children (Toronto, ON, Canada) for RNA sequencing. RNA sequencing was performed using the Illumina platform (paired-end reads of a 100 bp sequence), and mapped to the reference genome GRCM38 using HISAT2, as previously described [16]. The results were sent to Carleton University (Subedi) for further analysis. The pilot dataset was analyzed following the protocol from Pertea et al. [17] (the “new Tuxedo” package). The reads from the samples were mapped to the reference genome GRCM38 using HISAT2 [18]. All six samples had an overall alignment rate greater than 97%. The alignments were then passed to String Tie [17] for transcript assembly and quantification. The transcript abundance matrix from String Tie was then used for differential expression analysis using two different approaches: *edgeR* [19] and *DESeq2* [20]. The number of genes that had a *p*-value less than or equal to 0.05 were 606 and 1154 for *edgeR* and *DESeq2*, respectively (Supplementary Tables S1 and S2). Furthermore, the Benjamani–Hochberg procedure was used to adjust for multiple hypothesis testing [21] separately in the *edgeR* and *DESeq2* analyses. Using a threshold of 0.05 for the false discovery rate (FDR), 31 and 101 genes were identified as differentially expressed using *edgeR* and *DESeq2*, respectively (Supplementary Tables S3 and S4). Only genes with a known nomenclature are reported in this paper.

## 3. Results

**Figure 1:**
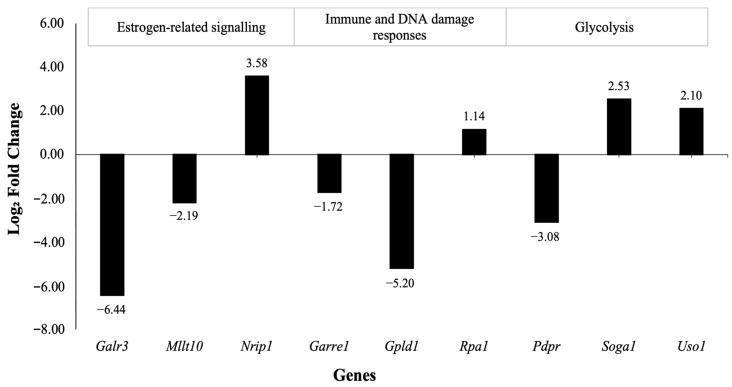
Comparison of concordant and differentially expressed genes (*p* ≤ 0.05) across both *edgeR* and *DESeq2* in isolated mammary epithelial cells of 6- to 8-week-old female transgenic *fat-1* mice (*n* = 3) relative to WT mice (*n* = 3). Data are *DESeq2*. See methods and details in Table 1.

Significant differences in the total RNA expression of 11 genes using *edgeR* (Table 1) and 45 genes using *DESeq2* (Table 2) were identified from the isolated mammary epithelial cells of *fat-1* mice compared to WT mice (adjusted *p* ≤ 0.05). Analysis using *edgeR* showed that there were five downregulated and six upregulated genes compared to WT mice (Table 1). For *DESeq2* analysis, there were 18 downregulated and 27 upregulated genes in the *fat-1* mice compared to WT mice (Table 2). Of these genes, nine were found to be significant across both the *edgeR* and *DESeq2* methods (adjusted *p* ≤ 0.05), where five genes were downregulated, and four genes were upregulated (Figure 1). The log2 fold changes for nine overlapping genes ranged from 1.14 to 7 (Figure 1).

A comparison of key pathways revealed distinct gene expression patterns between *fat-1* and WT mice. This study found that genes associated with estrogen-related signaling were stimulated with the downregulation of *mixed-lineage leukemia (Mllt10/AF10)* and a gene of *galanin receptor 3* (*Galr3)* and the upregulation of an essential marker of mammary gland development *nuclear receptor interacting protein 1* (*Nrip1/RIP140*) in *fat-1* mice compared to WT mice (Figure 1). The study also found that the changes in gene expression involved in glycolysis were upregulated, as indicated by the upregulation of *suppressor of glucose (Soga1)*, which is a negative regulator of gluconeogenesis, and *uso1 vesicle docking factor (Uso1)*, which is a regulator of insulin stimulus and the downregulation of *pyruvate dehydrogenase phosphatase regulatory subunit (Pdpr*) in *fat-1* mice compared with WT mice. On the other hand, there were effects in genes regulating immune and DNA damage responses with an upregulation in *granule associated rac and rhog effector* 1 (*Garre1)* and *replication protein a1* (*Rpa1)* along with a downregulation in *glycosylphosphatidylinositol specific phospholipase d1* (*Glpd1)* in *fat-1* mice compared to WT mice.

## 4. Discussion

This study has identified, in pubertal mammary epithelial cells, using RNA-Seq, differential expression of genes attributable to the presence or absence of *n*-3 PUFA in the transgenic *fat-1* mouse model. Using the transgenic *fat-1* mouse model [9,10], which is capable of de novo *n*-3 PUFA synthesis, this genetic approach provides evidence of within-cell gene expression changes attributable to *n*-3 PUFA. Thus, providing novel insight into how *n*-3 PUFA affects mammary gland development, a key lifecycle stage that potentially can reduce the development of mammary tumors.

At the onset of puberty, estrogen activity largely promotes the rapid growth and expansion of the ducts as TEBs invade to the edge of the fat pad to mature the ductal network [7]. Estrogen binds to its receptor ERα acting on the epithelium to sustain proliferation during ductal elongation [7]. Elevated prepubertal estrogen levels cause earlier puberty onset and increase the risk of BC potentially due to an increased number of proliferative TEBs [8]. Previous studies have demonstrated that prepubertal exposure to *n*-3 PUFA delays puberty onset, lowers estradiol and proliferation and reduces TEBs [9,10]. Consistent with these findings, this study found that *n*-3 PUFA decreases expression of the estrogen-responsive *Galr3*, which has been shown to increase five-fold with estradiol treatment in the female rat anterior pituitary gland [22]. While complete understanding of *Galr3* remains elusive, studies suggest *Galr3* exerts its function through Gi/Go to G proteins, leading to the inhibition of adenyl cyclase that perturbs the phosphorylation of CREB and is more expressed during proliferation of the mammary gland [29,30]. Thus, these changes in *Galr3* could suggest that *n*-3 PUFA contributes to reducing estrogen and proliferation. However, our lab more recently reported that lifelong *n*-3 PUFA exposure also increases ERα protein expression and the relative number of luminal mammary epithelial cells, suggesting additional effects on mammary epithelial cell differentiation [10]. Consistent with this finding, this study found that lifelong *n*-3 PUFA exposure decreases *Mllt10/AF10*, a cofactor of the disruptor silencing 1 like (DOT1L) responsible for di- and tri-histone H3-lysine 79 (H3K79) methylation [24,31]. One study found that H3K79 methylation depletes as mammary epithelial cells lose lineage commitment and become dedifferentiated [32]. While further investigation is required, this effect was hypothesized to occur through a hormonal and/or paracrine mechanism [32], which may be possible as a more recent study has shown that estrogen treatment enhances DOT1L and ERα interaction [33]. Consistent with this hypothesis, this study revealed a significant upregulation of *Nrip1*/*RIP140*, a critical estrogen-signaling mediator of ductal morphogenesis during pubertal mammary gland development [25]. A previous study reported that *RIP140* functions as a cofactor that is recruited with ERα to promote ERα-targeted gene expression [25]. The loss of *RIP140*, as seen in *RIP140* knockout mice, reduced luminal epithelial cells and impaired TEB formation during puberty [25]. While *RIP140* knockout mice were found to have lower numbers of TEBs than *RIP140* transgenic mice, due to impaired TEB formation, it was shown that overexpression of *RIP140* increases the number of alveolar buds that differentiate from TEBs [25]. Thus, these findings suggest that lifelong *n*-3 PUFA exposure increases estrogen-related signaling pathways under lower estrogen as a potential mechanism during pubertal mammary gland development.

While increasing estrogen-related signaling has also been shown to promote aerobic glycolysis in purified primary mammary mouse epithelial cells [34], limited evidence supports the hypothesis that *n*-3 PUFA increases the estrogen-related signaling that results in the promotion of glycolytic activity. Notably, a study in the triple-negative BC cell line MDA-MB-231 demonstrated that the *n*-3 PUFA, docosahexaenoic acid (DHA), decreased glycolytic activity and mitochondrial respiration [35]. In contrast, the authors also found that applying low concentrations of DHA (15 μM and 25 μM) to the human epithelial cell line of MCF-10A resulted in increased glycolytic utilization [35]. Consistent with these findings, this study shows that *n*-3 PUFA induces a glycolytic profile in healthy mammary epithelial cells during puberty. In mammary epithelial cells, lifelong *n*-3 PUFA exposure upregulated *Soga1*, which promotes glycolysis and reduces gluconeogenesis [27], and *Uso1*, which is highly expressed in glucose response [28], along with the downregulation of *Pdpr*, a gene involved in progressing acetyl-CoA into the citric acid cycle [36]. This glycolytic-like profile may align with evidence suggesting that *n*-3 PUFA can prolong the G1 phase of the cell cycle in embryonic stem cells, as glycolysis can occur during the G1 phase [37]. This halt in cell cycle progression in G1 has been reported to occur despite the presence of oxygen and functional mitochondria via aerobic glycolysis, also known as the Warburg effect [38,39]. More recently, the Warburg effect was found to occur not only in cancer cells but also uniquely in normal proliferating cells, maintaining intracellular pH during cell division [38]. Thus, these findings collectively suggest that *n*-3 PUFA could adaptively modulate metabolic activity in mammary epithelial cells differently in healthy and disease conditions, thus warranting further investigation.

The effects of *n*-3 PUFA mediating the immune and DNA damage response have been widely reported. Previous studies have suggested that *n*-3 PUFA have anticancer effects through the immune system, such as by suppressing CD4+ T-cell activation and reorganizing cell signaling [40]. Consistent with this, we found that lifelong *n*-3 PUFA exposure downregulates *Glpd1*, a phospholipase D1 glycosylphosphatidylinositol anchor, in which splenic CD4+ T cells were found to be downregulated in phospholipase D1 (PLD1) knockout mice [23]. Furthermore, phospholipase D inhibition was shown to reduce BC invasion by lowering PLD1 [23], which is consistent with a study that shows that treatment with essential *n*-6 PUFA increases PLD1 and BC invasion in vitro [41]. However, we also observed an upregulation in *Garre1*, a master regulator of the CCR4-Not complex binding activity with critical roles in the immune system [42,43,44]. The diverse functions of the CCR4-Not complex, such as cell cycle control, chromatin modification, and transcription activity, allow for the rapid adaption of gene expression in response to environmental changes [42]. A previous study showed that the CCR4-Not complex regulates genomic stability [43], making it a target of interest for cancer therapies [44]. In tandem, we also found that *n*-3 PUFA *Rpa1*, which supports genomic integrity with essential roles in DNA replication, recombination, and repair [26], is decreased in HER2-positive BC [45]. *Rpa1* is also highly expressed in the immune system of mice, particularly in the lymph nodes and spleen, where it plays a crucial role in maintaining T-cell homeostasis [46]. Thus, these findings suggest that *n*-3 PUFA could have critical effects on mammary epithelial cells that are important for maintaining immune function during development, warranting further investigation.

This pilot study has strengths and limitations that should be considered. These results are exclusive to total mammary epithelial cells in 6- to 8-week-old female *fat-1* mice, which consist of both luminal and myoepithelial cells. This study also had a relatively small sample size of three per group. Nevertheless, we used two robust bioinformatic techniques appropriate for small sample sizes [47]. Additionally, the oestrous stage of each mouse was not determined, which could add variability to the results due to higher progesterone and cell proliferation levels during diestrus [48]. Lastly, this study utilized total RNA, including both mRNA and non-coding RNA; however, total RNA has the largest gene library available for clinical use [49,50]. Despite these limitations, this study demonstrated the utility of determining cell-specific changes in gene expression by RNA-Seq. Further, the use of the *fat-1* model made it possible to study the effects of *n*-3 PUFA on mammary gland development without the potential confounding effects of dietary intake [9,10].

## 5. Conclusions

In conclusion, this study advances our fundamental knowledge of the role of *n*-3 PUFA in pubertal mammary gland development in female *fat-1* mice utilizing cutting edge RNA-Seq technology and bioinformatics approaches. These findings suggest that lifelong *n*-3 PUFA exposure may have long-term protective effects for BC prevention mediated at the level of mammary epithelial cells. Therefore, future studies are warranted to investigate the effects of *n*-3 PUFA on mammary gland development within specific cell types, leading to a better understanding of how diet contributes to reduced BC risk later in life.

## Figures and Tables

**Table 1:** Effects of lifelong *n*-3 polyunsaturated fatty acid exposure on gene expression in isolated pubertal mammary epithelial cells between *fat-1* and WT mice assessed by *edgeR*.

Gene	Log_2_ Fold Change	Adjusted *p*-Value	Function(s):	NIH National Library of Medicine Gene ID
*edgeR*				
*Galr3* *, galanin receptor 3	−7.00	<0.0001	Positive regulation of transcription by RNA polymerase II, G-protein-coupled receptor signaling pathway, peptide hormone binding activity, estrogen response marker [22]	14429
*Garre1*, granule associated rac and rhog effector 1	−1.71	<0.0001	Enables CCR4-Not complex binding activity and Rac signaling production	233103
*Gpld1* *, glycosylphosphatidylinositol-specific phospholipase d1	−5.18	<0.0001	Enables phospholipase D activity, sodium channel regulator, positive regulator of HDL particle clearance, positive regulation of insulin secretion in response to glucose, regulation of triglyceride metabolic process, immune cell recruitment [23]	14756
*Mllt10* (AF10) *, mixed-lineage leukemia	−2.19	<0.0001	Enables histone and nucleosome binding, positive regulation of transcription by RNA polymerase II, H3K79 methylation regulation by DOT1L [24]	17354
*Nrip1 (RIP140)* *, nuclear receptor interacting protein 1	3.58	<0.0001	Negative regulator of transcription by RNA polymerase II, Circadian rhythm, lipid storage, histone deacetylase complex, retinoid X receptor binding activity. Role in estrogen signaling and mammary gland development [25]	268903
*Pdpr* *, pyruvate dehydrogenase phosphatase regulatory subunit	−3.08	<0.0001	Enables oxidoreductase activity	319518
*Rpa1* *, replication protein a1	1.14	<0.0001	Enables chromatin binding activity, DNA repair, DNA replication, DNA recombination [26]	68275
*Slc24a2*, solute carrier family 24 member 2	3.72	<0.0001	Enables calcium channel activity	76376
*Soga1* *, suppressor of glucose	2.53	<0.0001	Insulin receptor signaling, macronutrient metabolism, negative regulation of gluconeogenesis, regulation of autophagy [27]	N/A
*Terc*, telomerase rna component	2.80	<0.0001	Involved in telomere maintenance and chromosomal repair	21748
*Uso1*, uso1 vesicle docking factor	2.10	<0.0001	Regulation of insulin stimulus, secretory granule localization, small GTPase signal transduction, intracellular protein transport, marker of glucose utilization [28]	56041

Data are log_2_ fold change. Change in total RNA expression within isolated total mammary epithelial cells of transgenic *fat-1* mice, which genetically reflects lifelong *n*-3 polyunsaturated fatty acid (*n*-3 PUFA) exposure [10], compared to female wildtype (WT) mice on a *n*-6 polyunsaturated fatty acid (*n*-6 PUFA)-rich diet aged 6 to 8 weeks. Three pooled samples from *fat-1* and WT mice underwent RNA sequencing, performed using the Illumina platform (paired-end reads of a 100 bp sequence), and mapped to reference genome GRCM38 using HISAT2 [18]. Differential expression analysis was performed using *edgeR* followed by multiple testing correction using the Benjamani–Hochberg procedure to control for the false discovery rate (FDR) at 5% [21]. Using a threshold of an FDR-adjusted *p*-value of 0.05 revealed 31 genes that were differentially expressed by *edgeR*. Of these, 11 genes with an known nomenclature are reported in this Table. * Genes that were identified as differentially expressed compared to the WT using the edgeR method after Benjamani–Hocheburgh adjustment for multiple hypothesis testing. Gene functions were found using Gene ID description from the NIH National Library of Medicine on 4 November 2023. References were provided for additional gene functions not listed in the NIH National Library of Medicine.

**Table 2:** Effects of lifelong *n*-3 polyunsaturated fatty acid exposure on gene expression in isolated pubertal mammary epithelial cells between *fat-1* and WT mice assessed by *DESeq2*.

Gene	Log_2_ Fold Change		Adjusted *p*-Value	Function(s):	NIH National Library of Medicine Gene ID
*DESeq2*					
*Arfip1*, adp ribosylation factor interacting protein 1	−1.33	<0.0001	Enables phosphatidylinositol-4-phosphate binding activity; negative regulator of retrograde transport	99889	
*Arl4a*, adp ribosylation factor-like gtpase 4	1.10	<0.0001	Enables GTP binding activity	11861	
*B3gat3*, beta-1,3-glucuronyltransferase 3	1.79	0.01	Enables galactosylgalactosylxylosylprotein 3-beta-glucuronosyltransferase activity and protein phosphatase activator activity	72727	
*Baz2b*, bromodomain adjacent to zinc finger domain 2b	−0.84	0.001	Enables DNA binding activity; Chromatin remodeling	407823	
*Chchd2-ps*, coiled-coil-helix-coiled-coil-helix domain containing 2	1.61	0.046	N/A	433806	
*Clpp*, caseinolytic mitochondrial matrix peptidase proteolytic subunit	1.10	0.001	Enables ATP-dependent peptidase; membrane protein proteolysis and protein quality control	53895	
*Csf2rb2*, colony stimulating factor 2 receptor subunit beta	−2.96	0.036	Enables cytokine receptor activity; cytokine-mediated signaling	12984	
*Cspp1*, centrosome and spindle pole-associated protein 1	−0.48	0.0004	Positive regulator of cytokinesis	211660	
*Dhcr7*, 7-dehydrocholesterol reductase	−4.23	0.03	Enables 7-dehydrocholesterol reductase activity (lipid metabolism)	13360	
*Dohh*, deoxyhypusine hydroxylase	1.16	0.0001	Enables deoxyhypusine monooxygenase activity and iron ion binding activity; acts upstream of or within peptidyl-lysine modification to peptidyl-hypusine	102115	
*Galr3* *, galanin receptor 3	−6.44	0.002	Positive regulation of RNA polymerase II; G-protein-coupled receptor signaling pathway; peptide hormone binding activity; estrogen response marker [22]	14429	
*Garre1* *, granule-associated rac and rhog effector 1	−1.72	<0.0001	Enables CCR4-Not complex binding activity; Rac signaling	233103	
*Gigyf1*, grb10 interacting gyf protein 1	−0.82	<0.0001	Involved in insulin-like growth factor receptor signaling	57330	
*Glg1*, golgi apparatus protein 1	−0.57	<0.0001	Enables cell surface interactions; fibroblast growth factor binding activity; negative regulator of beta receptor signaling pathway	20340	
*Gpatch8*, g-patch domain containing 8	1.42	0.0004	Enables metal ion activity and nucleic acid binding activity	237943	
*Gpld1* *, glycosylphosphatidylinositol-specific phospholipase d1	−5.20	<0.0001	Enables phospholipase D activity; sodium channel regulator; positive regulator of HDL particle clearance; positive regulation of insulin secretion in response to glucose; regulation of triglyceride metabolic process, immune cell recruitment [23]	14756	
*Hnrnpl*, heterogeneous nuclear ribonucleoprotein l	0.51	0.003	Enables mRNA-binding protein activity; regulator of alternative mRNA splicing	15388	
*Map3k4*, mitogen-activated protein kinase 4	5.05	0.0003	Enables protein kinase activity; positive regulator of JUN kinase activity	26407	
*Med8*, mediator complex subunit 8	0.67	<0.0001	Enables RNA polymerase II cis-regulator region DNA binding activity	80509	
*Mllt10* (*AF10*) *, mixed-lineage leukemia	−2.19	0.002	Enables histone and nucleosome binding; positive regulator of transcription by RNA polymerase II, regulating H3K79 methylation by DOT1L [24]	17354	
*Morc4*, morc family cw-type zinc finger 4	0.90	0.007	Enables methylated histone binding activity	75746	
*Nrip1 (RIP140)* *, nuclear receptor interacting protein 1	3.58	<0.0001	Negative regulator of transcription by RNA polymerase II, Circadian rhythm, lipid storage, histone deacetylase complex, retinoid X receptor binding activity Role in estrogen signaling and mammary gland development [25]	268903	
*Pbxip1*, pbx homeobox interacting protein 1	1.80	0.02	Enables transcription coactivator activity, histone H3-K56 acetylation, extracellular matrix organization	229534	
*Pcdha9*, protocadherin alpha 9	6.23	0.011	Enables calcium binding activity; involved in cell adhesion	192161	
*Pdpr* *, pyruvate dehydrogenase phosphatase regulatory subunit	−3.08	0.0002	Enables oxidoreductase activity	319518	
*Per1*, period circadian regulator 1	−0.69	0.008	Involved in circadian rhythm	18626	
*Phc3*, polyhomeotic homolog 3	−2.06	0.03	Negative regulator of transcription, PcG protein complex, chromatin binding activity, histone binding activity	241915	
*Plin3*, perilipin 3	3.67	0.013	Involved in lipid storage and positive regulator of sequestering of triglyceride	66905	
*Pnp*, purine nucleoside phosphorylase	−1.64	0.023	Enables guanosine phosphorylase activity and purine nucleoside phosphorylase activity	18950	
*Prdm11*, pr/set domain 11	−1.05	0.042	Enables chromatin binding activity; negative regulator of cell growth	100042784	
*Rapgef3*, rap guanine nucleotide exchange factor 3	−0.38	0.033	Enables guanyl-nucleotide exchange factor activity.	223864	
*Rbm26*, rna binding motif protein 26	0.95	0.0003	Enables RNA binding activity; involved in mRNA processing	74213	
*Rn7s1*, 7s rna	4.30	0.002	N/A	103948	
*Rn7s6*, 7s rna- 6	5.18	0.001	N/A	109568	
*Rpa1* *, replication protein a1	1.14	<0.0001	Enables chromatin binding activity, DNA repair, DNA replication, DNA recombination [26]	68275	
*Rrp8*, ribosomal rna processing 8	1.93	0.036	Enables methylated histone binding activity; involved in cellular response to glucose starvation, intrinsic apoptotic signaling pathway by p53 class mediator, chromosome organization	101867	
*Sfr1*, swi5-dependent homologous recombination repair protein 1	0.56	0.007	Enables nuclear receptor coactivator activity, double-stand break repair, cell cycle, DNA repair	67788	
*Soga1* *, suppressor of glucose	2.53	0.008	Insulin receptor signaling, macronutrient metabolism, negative regulation of gluconeogenesis, regulation of autophagy [27]	N/A	
*Stard9*, star-related lipid transfer domain containing 9	2.14	0.046	Enables ATP hydrolase activity; involved in microtube movement and spindle assembly	668880	
*Tbc1d10a*, tbc1 domain family member 10a	0.91	<0.0001	Enables GTPase activator activity and PDZ domain binding activity; positive regulator of hydrolase activity; involved in protein transport	103724	
*Tmc8*, transmembrane channel like 8	−1.72	<0.0001	Enables TNF-alpha binding activity, zinc homeostasis; regulation of extrinsic apoptosis signaling death receptors	217356	
*Tpt1-ps3*, tumor protein, translationally controlled	4.17	0.005	Cell growth and proliferation	100043703	
*Uso1* *, uso1 vesicle docking factor	2.10	<0.0001	Regulation of insulin stimulus, secretory granule localization, small GTPase signal transduction, intracellular protein transport, glucose response marker [28]	56041	
*Vps13b*, vacuolar protein sorting 13 homolog b	0.84	<0.0001	Involved in protein transport	666173	
*Xpo4*, exportin 4	1.07	<0.0001	Enables nuclear export signal receptor activity	57258	

Data are log_2_ fold change. Change in total RNA expression within isolated total mammary epithelial cells of transgenic *fat-1* mice, which genetically reflects lifelong *n*-3 polyunsaturated fatty acid (*n*-3 PUFA) exposure [10], compared to female wildtype (WT) mice on a *n*-6 polyunsaturated fatty acid (*n*-6 PUFA)-rich diet aged 6 to 8 weeks. Three pooled samples from *fat-1* and WT mice underwent RNA sequencing, performed using the Illumina platform (paired-end reads of a 100 bp sequence), and mapped to reference genome GRCM38 using HISAT2 [18]. Differential expression analysis was performed using *DESeq2* followed by multiple testing correction using the Benjamani–Hochberg procedure to control for false discovery rate (FDR) at 5% [21]. Using a threshold of the FDR-adjusted *p*-value of 0.05 revealed 101 genes that were differentially expressed by *DESeq2*. Of these, 45 genes with a known nomenclature are reported in this Table. * Genes that were identified as differentially expressed compared to the WT using the DeSeq2 method after the Benjamini–Hocheburgh adjustment for multiple-hypothesis testing. Gene functions were found using the Gene ID description from the NIH National Library of Medicine on 4 November 2023. References were provided for additional gene functions not listed in the NIH National Library of Medicine.

## Data Availability

The authors declare that all data supporting the study findings are within the article, and the RNA sequencing data have been deposited in the Gene Expression Omnibus (GEO) database under accession code GSE281867 (Available online: https://www.ncbi.nlm.nih.gov/geo/query/acc.cgi?acc=GSE281867 (accessed on 14 November 2024).

## Supplementary Materials

The following supporting information can be downloaded at: https://www.mdpi.com/article/10.3390/nu16223925/s1.
